# Effects of controlled abnormal joint movement on the molecular biological response in intra-articular tissues during the acute phase of anterior cruciate ligament injury in a rat model

**DOI:** 10.1186/s12891-018-2107-6

**Published:** 2018-05-29

**Authors:** Yuichi Nishikawa, Takanori Kokubun, Naohiko Kanemura, Tetsuya Takahashi, Masayasu Matsumoto, Hirofumi Maruyama, Kiyomi Takayanagi

**Affiliations:** 1 Department of Neuroscience and Therapeutics, Hiroshima University Graduate School of Biomedical and Health Sciences, Hiroshima, Japan; 2 Division of Rehabilitation, Department of Clinical Practice and Support, Hiroshima University Hospital, Hiroshima, Japan; 3 Department of Physical Therapy, School of Health and Social Services, Saitama Prefectural University, 820 Sannomiya, Koshigaya-shi, Saitama, 343-8540 Japan; 4 Sakai City Medical Center, Osaka, Japan

**Keywords:** Anterior cruciate ligament, Matrix metalloproteinase-13, Conservative therapy, Acute phase injury

## Abstract

**Background:**

The anterior cruciate ligament (ACL) is responsible for braking forward movement of the tibia relative to the femur and for tibial rotation. After ACL injury, this braking performance deteriorates, inducing abnormal joint movement. The purpose of this study was to clarify the effects of controlled abnormal joint movement on the molecular biological response in intra-articular tissues during the acute phase of ACL injury.

**Methods:**

Eighty-four mature Wistar male rats were randomly assigned to a controlled abnormal movement (CAM) group, an ACL-transection (ACL-T) group, a sham-operated group, or an intact group. The ACL was completely transected at its midportion in the ACL-T and CAM groups, and a nylon suture was used to control abnormal tibial translation in the CAM group. The sham-operated group underwent skin and joint capsule incisions and tibial drilling without ACL transection. Animals were not restricted activity until sacrifice 1, 3, or 5 days after surgery for histological and gene expression assessments. Acute-phase inflammation requires an important balance between degenerative and biosynthetic processes and is controlled by the activities of matrix metalloproteinases (MMPs) and tissue inhibitors of metalloproteinases (TIMPs). Both types of gene were analyzed in this study.

**Results:**

The ACL-T and CAM groups exhibited cleavage of the ACL at all time points. However, for the CAM group, the gap in the ligament stump was extremely small, and fibroblast proliferation was observed around the stump. Relative to the ACL-T group, the CAM group demonstrated significantly lower expression of MMP-13 mRNA and a lower MMP-13/TIMP-1 ratio on days 1 and 5 in the ACL, the medial meniscus and the lateral meniscus. The expression of TIMP-1 mRNA was not significantly different between the ACL-T and CAM groups.

**Conclusions:**

The study results suggested that controlling abnormal movement inhibited the inflammatory reaction in intra-articular tissues after ACL injury. This reaction was down-regulated in intra-articular tissues in the CAM group. Abnormal joint control caused prolonged inflammation and inhibited remodeling during the acute phase of ACL rupture.

## Background

The anterior cruciate ligament (ACL) plays an important role in controlling and stabilizing the knee joint; it is the primary restraint against anterior tibial translation [1]. The prevalence of injury to the ACL is quite high, particularly among athletes who perform pivoting activities [2, 3]. Furthermore, previous reported that female subjects significantly higher risk of ACL injury than male subjects [4, 5]. Treatment after ACL injury often involves reconstructive surgery, which is performed on more than half of ACL injury patients [6, 7].

ACL deficiency can induce the degeneration of other intra-articular tissues (i.e., cartilage and meniscus), which is a risk factor for the development of osteoarthritis [8]. Previous studies have generally attributed injury-induced knee degeneration to the long-term biomechanical changes in the microenvironment of the knee joint and have primarily focused on the long-term molecular kinetics in injured ACLs [9, 10]. Consequently, many studies have shown that meniscal damage and chondral degeneration occur with chronic ACL deficiency [11–13]. Furthermore, previous studies have reported that ACL blood supply is poor [14] and he lack of a scaffold [15]. Therefore, ACL is recognized as a ligament difficult to heal after injury.

Surgical reconstruction treatment is the standard treatment after ACL rupture. ACL reconstruction is the best choice for athletes and/or high-level activity patients. However, conservative therapy after ACL injury is selected for patients with low and/or moderate activity levels, children, elderly people. Although the ACL is not known to heal spontaneously in general [16], there are many previous reports documenting spontaneous healing of a ruptured ACL [15, 17–25]. Ihara et al. reported that 3-month conservative treatment resulted in a well-defined, normal-sized, straight band in 74% of patients with complete ACL rupture [23]. Moreover, many studies have experimentally demonstrated the functional healing responses of injured ACLs [15, 17–19]. Extra-articular ligaments such as the medial collateral ligament (MCL) exhibit a well-described healing response after injury in the absence of surgical procedures [26]. Nguyen et al. showed that the human proximal 1/3 ACL has an intrinsic healing response with typical histological characteristics similar to those of the MCL [15]. Although these studies [15, 17–19] have reported that the ACL remnant has some possible functional healing responses that may induce spontaneous healing, this theory has not been confirmed.

A previous study demonstrated the effects of controlled abnormal joint motion on modifying the intra-articular molecular response of ACL-ruptured knees, which led to spontaneous ACL healing [27]. Although that study demonstrated a new mechanism of ACL healing, the molecular biological responses of the intra-articular tissues during the ACL healing process in the acute phase of injury remain unclear. It is thought that controlled abnormal joint movement is an important factor for spontaneous ACL healing. Many previous studies have reported the molecular biological responses in the intra-articular tissues during the acute phase [28–31]. However, to the best of the authors’ knowledge, no reports have yet focused on the effects of abnormal joint movement on intra-articular tissues. The acute-phase inflammatory response plays an important role in the wound healing process, and the balance between the degenerative and biosynthetic arms of this process is controlled by the activities of matrix metalloproteinases (MMPs) and tissue inhibitors of metalloproteinases (TIMPs) [32]. In the present study, we focused on MMPs and TIMPs to determine the molecular biological response in the intra-articular tissues during acute-phase ACL injury.

The objective of the present study was to elucidate the effects of controlled abnormal joint movement on the molecular biological response in intra-articular tissues during the acute phase of ACL injury. We hypothesized that controlling abnormal joint movement in a rat model would decrease the inflammatory response in intra-articular tissues after the ACL injury acute phase.

## Methods

### Experimental design

**Fig. 1:**
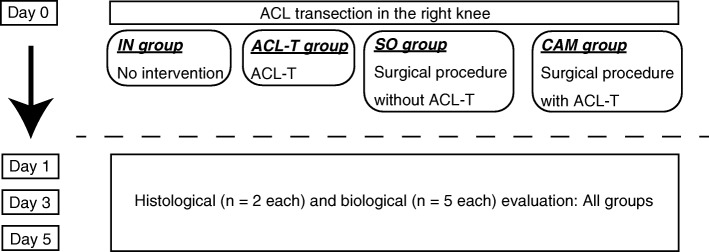
Flowchart showing the allocation of animals in the study. ACL-T, anterior cruciate ligament transection; CAM, controlled abnormal movement; IN, intact; SO, sham operated. Each group, *n* = 21

All experiments were approved by the Saitama prefectural University Animal Experiment Ethics Committee (permit no. 24–2), and performed in accordance with their Guidelines for the Care and Use of Laboratory Animals. Eighty-four mature, 12-week-old Wistar male rats (body weight, 380–428 g, Japan SLC, Shizuoka, Japan) were housed individually on a 12-h light-dark cycle with free access to food and water. The male rats were randomly assigned to the controlled abnormal movement (CAM) groups, ACL-transection (ACL-T), sham-operated (SO), or intact (IN), (each group, *n* = 21). The animals were not restricted activity until sacrifice. The room temperature was maintained at 23 °C ± 2 °C. To determine the inflammatory response, the ACL and meniscus of the CAM group were histologically compared with those of the other groups 1, 3, and 5 days after surgery (for 2 rats from each group); the inflammatory response was also compared across groups at 1, 3, and 5 days after surgery (for 5 rats from each group) (Fig. 1).

### Surgical procedure

**Fig. 2:**
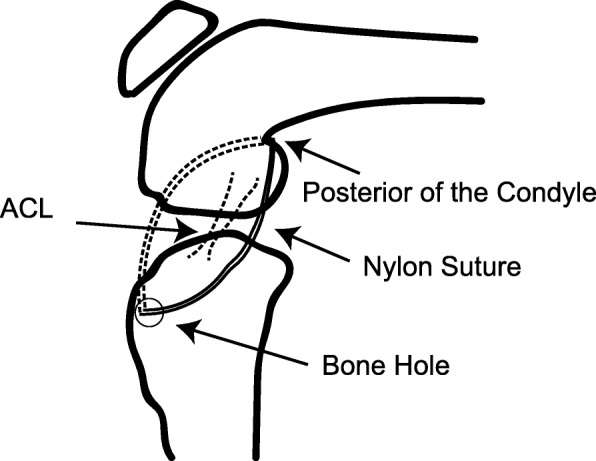
Extra-articular braking model. In the CAM group, a 2–0 nylon suture was passed through the tibial bone tunnel posterior to the condyle of the distal end of the femur to control anterior tibial translation and was then tied to the joint to prevent abnormal tibial translation without restriction of knee flexion. ACL, anterior cruciate ligament. CAM, controlled abnormal movement

Prior research showed that changing the joint kinematics of a knee with complete ruptured ACL results in the down-regulation of inflammatory responses [33] and leads to spontaneous healing [27]. These previous studies used the CAM model used for the purpose of controlling abnormal knee joint movement without restricted knee flexion (Fig. 2). Unlike casts, the CAM model restricts only the anterior drawer of the tibia, therefore it does not inhibit the flexion of the knee joint. This model was devised with reference to the brace used in previous study [23]. These previous studies showed that controlling the motion of the knee and minimizing abnormal sagittal deviations between the femur and tibia within the range of motion appropriate for ACL injury treatment leads to spontaneous healing [22, 23]. Therefore, in the present study, this CAM model was used to determine the effect of changing the intra-articular environment during the acute phase of ACL injury.

The animals were anesthetized with pentobarbital (10 mg/kg) via intraperitoneal injections. The right knee underwent medial parapatellar arthrotomy, and the ACL was horizontally and completely transected at the midportion. After the ACL was transected, the joint capsule and parapatellar fascia were closed with a running suture using 4–0 Ethibond (Ethicon Endo-Surgery Japan, Tokyo, Japan), and a bone tunnel was created in the medial aspect of the tibial tuberosity in the mediolateral direction. Then, the skin was closed only in the ACL-T group. In the CAM group, a 2–0 nylon suture (Prolene, Ethicon Endo-Surgery Japan) was passed through the tibial bone tunnel posterior to the condyle of the distal end of the femur and was then tied to the joint in order to prevent abnormal tibial translation, according to the same procedure as that used in previous study [27]. The nylon suture provided a directed traction force to resist the anterior motion of the tibia without restricted knee flexion (Fig. 2). After extra-articular braking, the skin was closed with running sutures. The SO group underwent skin and joint capsule incisions and tibial bone tunnel creation without ACL transection; postoperatively, these rats were immediately allowed unrestricted movement.

### Histological examination

The intra-articular response in the acute phase of ACL injury was evaluated histologically at 1, 3, and 5 days, according to the same procedure as that used in previous study [27]. Two animals (each group) were sacrificed by exsanguination, and fixed in 4% paraformaldehyde after anesthetized with pentobarbital (10 mg/kg) via intraperitoneal injections at 1, 3, and 5 days, and all tissues were decalcified in a 10% ethylenediaminetetraacetic acid-based solution (pH 7.4) at 4 °C for 5 to 6 weeks. After decalcified, the all tissues were infiltrated with phosphate buffered saline different containing sucrose at 4 °C (10%; 4 h, 15%; 4 h, and 20%;12 h), and embedded in an optimal cutting temperature compound (O.C.T., Sakura Finetek Japan, Tokyo, Japan). Longitudinal cryosections were cut along the sagittal plane with a thickness of 14 μm using cryostat (Leica 3050 S, Leica Microsystems AG, Wetzlar, Germany) and maintained at − 80 °C. The cryosections were stained with hematoxylin and eosin (H&E) in order to observe the microscopic morphological characteristics of the intra-articular response in the acute phase of ACL injury.

### Molecular biological evaluation

On postoperative days 1, 3, and 5, ACLs and menisci were harvested from each group (*n* = 5) and evaluated gene expression related to the intra-articular reaction using real-time reverse transcription polymerase chain reaction (PCR). The tissue samples were homogenized, and RNA was extracted using an Allprep DNA/RNA/Protein mini kit (Qiagen, Hilden, Germany). Total RNA from each sample was reverse transcribed into complementary DNA (cDNA) using a high-capacity RNA to cDNA kit (Applied Biosystems, CA, USA), according to the same procedure as that used in previous study [27]. Real-time PCR was performed using a Chrome 4 Real-Time Detector (Bio-Rad Laboratories, Hercules, USA) with TaqMan Gene Expression Assay probe inflammatory factors, matrix metalloproteinase-13 (MMP-13), and tissue inhibitor of metalloproteinase-1 (TIMP-1), according to the manufacturer’s instructions (Applied Biosystems). Beta-actin was selected as the reference gene. The primers used are listed in Table 1 (TaqMan Gene Expression Assay, Applied Biosystems).

**Table 1:** Gene expression assays used for real-time PCR

Gene	Assay number
Matrix metalloproteinase-13 (MMP-13)	Rn01448194
Tissue inhibitor of metalloproteinase-1 (TIMP-1)	Rn00580432
Beta-actin	Rn00667869_m1

Standard curves were established with standards prepared from 1st standard cDNA (Genostaff, Tokyo, Japan) for all primers. The transcript levels of the target genes were normalized to beta-actin.

### Statistical analysis

The experimental data are presented as the mean ± standard deviation (SD). Before analysis, the normal distribution of the data was confirmed using the Shapiro-Wilk test. Gene expression was analyzed using two-way (group x time point) analysis of variance (ANOVA), with subsequent post hoc comparisons (via Bonferroni tests) used for comparisons among groups and time points (i.e., days 1, 3, and 5). A *p value* of less than 0.05 was considered significant. Statistical analyses were performed using JMP statistical software, ver. 12.0 (SAS Institute, Inc., Cary, NC, USA).

## Results

### Influence of intra-articular tissues after ACL transection

**Fig. 3:**
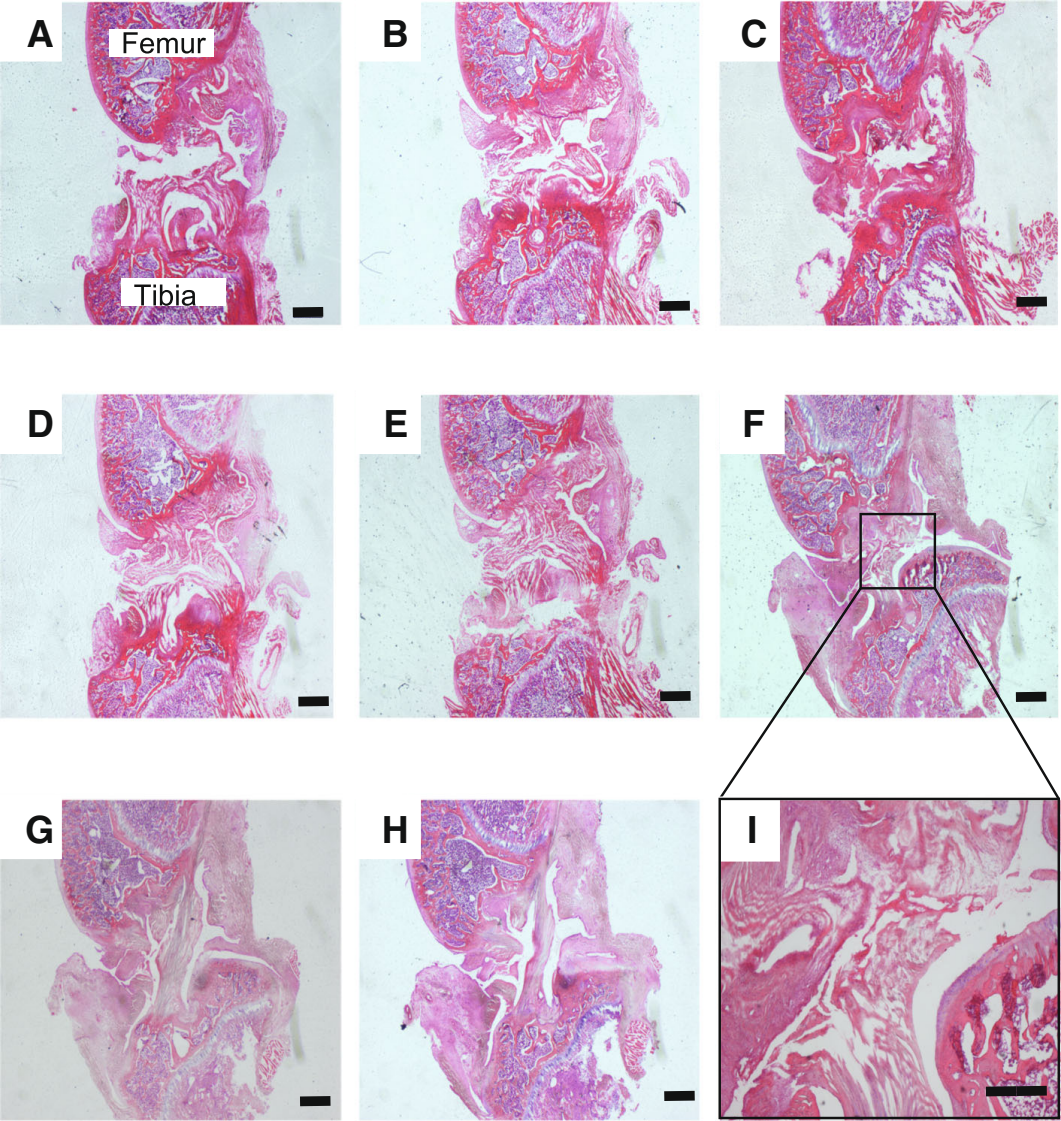
Histological evaluation in each group. These figures show longitudinal sections of the anterior cruciate ligament (ACL) stained with hematoxylin and eosin: (**a**) ACL-T group at day 1; (**b**) ACL-T group at day 3; (**c**) ACL-T group at day 5; (**d**) CAM group at day 1; (**e**) CAM group at day 3; (**f**, **i**) CAM group at day 5; (**g**) IN group; (**h**) SO group. The ACL-T and CAM groups demonstrated cleavage of the ACL at each time point (**a**–**f**). However, the gap in the ligament stump in the CAM group became very small, and fibroblast proliferation was observed around the stump (**d**–**f**, **i**). ACL-T, ACL-transection; CAM, controlled abnormal movement; IN, intact; SO, sham operated. Scale bars = 1 mm

The ACL-T and CAM groups demonstrated cleavage of the ACL at days 1, 3 and 5 (Fig. 3a–f). In the ACL-T and CAM group, ACL cleavage could be confirmed in all tissue (Fig. 3a–f and i). Five days after operation, histology showed widening of the gap of the ligament stump in the ACL-T group (Fig. 3c). In the CAM group, the gap of the ligament stump decreased (Fig. 3f and i). In the SO and IN groups, the ACL showed continuity based on the arrangement of the collagen fibers (Fig. 3g and h).

### Expression of MMP-13 and TIMP-1 mRNA in intra-articular tissues after ACL injury

**Fig. 4:**
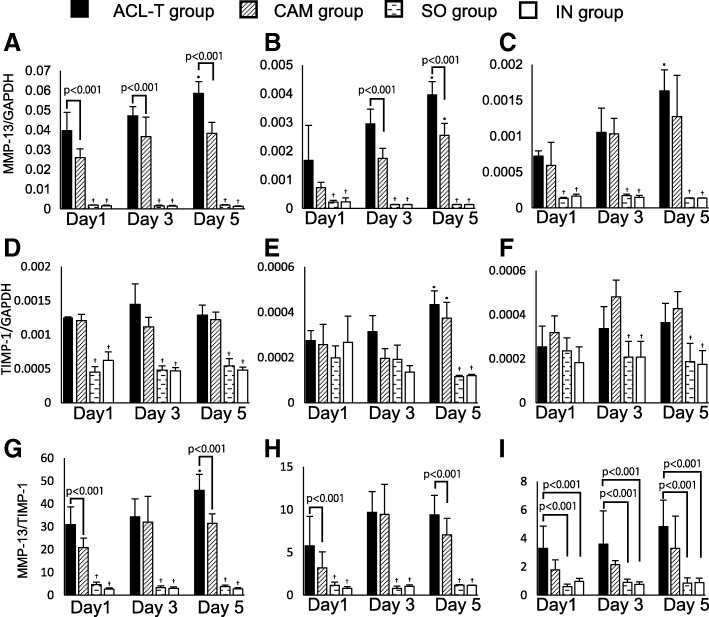
Gene expression at days 1, 3, and 5 after surgery. Expression levels of matrix metalloproteinase-13 (MMP-13) in the anterior cruciate ligament (ACL, **a**), medial meniscus (**b**), and lateral meniscus (**c**). MMP-13 expression in the ACL and medial meniscus differed significantly between the ACL-T and CAM groups. The expression levels of tissue inhibitor of metalloproteinase-1 (TIMP-1) in the ACL (**d**), medial meniscus (**e**), and lateral meniscus (**f**). TIMP-1 in all intra-articular tissues was not significantly different between the ACL-T and CAM groups. MMP-13/TIMP-1 ratios in the ACL (**g**), the medial meniscus (**h**), and the lateral meniscus (**i**). The ACL-T and CAM groups significantly differed with respect to the MMP-13/TIMP-1 ratio in the ACL and the medial meniscus on days 1 and 5 after surgery. On the other hand, in the lateral meniscus, the MMP-13/TIMP-1 ratio was significantly higher for the ACL-T group than for the other groups, with no significant difference between the CAM group and the SO and IN groups. ACL-T, ACL-transection; CAM, controlled abnormal movement; SO, sham operated, * *p* < 0.001 compared with day 1, † *p* < 0.001 compared with the ACL-T and CAM groups. Data showed mean ± standard deviation

Significant differences in mRNA levels for MMP-13 in the ACL and medial meniscus were observed during the acute phase ACL injury between ACL-T and CAM groups (*p* < 0.0001, Fig. 4a, b). Furthermore, the all intra-articular tissues showed a significantly higher expression on day 5 than day 1 in the ACL-T group (Fig. 4a–c). The CAM group showed a significantly higher expression on day 5 than day 1 only medial meniscus. On the other hands, there was no significant difference in TIMP-1 mRNA expression between the ACL-T group and the CAM group (*p* = 0.384, Fig. 4d–f). As with MMP-13 mRNA in the MMP-13/TIMP-1 ratio, a significant difference was observed in the ACL and medial meniscus between ACL-T and CAM groups (*p* < 0.01, Fig. 4g and h). In the lateral meniscus, the MMP-13/TIMP-1 ratio was significantly higher in the ACL-T group than that in the SO and IN groups at each time point (*p* < 0.001 for all comparisons, Fig. 4i). The CAM group did not significantly differ from the SO and IN groups with respect to the MMP-13/TIMP-1 ratio at each time point. The SO and IN groups showed significant differences in each gene expression in any intra-articular tissues compared with the ACL-T and CAM groups (except the medial meniscus at day 1 and 3, the lateral meniscus at day 1in TIMP-1 mRNA, and lateral meniscus at each time point in MMP-13/TIMP-1 ratio).

## Discussion

The present study was to compare the intra-articular response between the CAM and ACL-T groups during the acute phase of ACL injury using histological and gene expression method. The primary results of the present study are the following: as compared with ACL-T group, CAM group exhibited (1) the gap of the ligament stump decreased, (2) significantly lower MMP-13/TIMP-1 expression ratios. These findings partially supported our hypothesis; the inflammatory reaction in the intra-articular tissues decreased in the CAM group, and joint control was associated with the inflammatory reaction during the acute phase of ACL injury.

Our previous study demonstrated the effects of controlled abnormal joint motion on modifying the intra-articular molecular response of ACL ruptured knees, which led to spontaneous ACL healing [27]. Thus, our previous study showed spontaneous ACL healing at 2 weeks postoperative using the controlled abnormal joint motion procedure. In the present study, a rat model of this spontaneous healing was used to determine the effect of changing the intra-articular environment in a CAM model during the acute phase of ACL injury.

In the present study, controlled abnormal tibial translation led to a decrease in the regression of the ligament stump and a decrease in the inflammatory reaction in the intra-articular tissues during acute-phase ACL injury. In contrast, the ACL-T group showed degeneration of the ligament remnant. The difference between the ACL-T and CAM groups is the presence or absence of controlling abnormal joint movement. In general, failing to control abnormal joint movement prevents healing after ACL injury. A previous study reported that partial ACL injuries cause 42% of patients to develop complete ACL insufficiency [34]. Consequently, abnormal joint movement was degenerative for the ACL remnant. Furthermore, the poor healing capacity of the ACL has been noted both experimentally and clinically (e.g., there are issues with vascular supply [14], the lack of a scaffold [15], and the lack of blood clot formation [35]). A previous study showed that complete rupture of the ACL, showing a “mop end” of the remnant in the inflammatory phase, led to gradual retraction of the ligament remnant [18]. These previous findings are in accordance with the results of the present study showing that the ACL-T group exhibited retraction of the ligament remnant. However, Ihara et al. showed that spontaneous healing of the ACL occurs upon conservative treatment with early protective mobilization after complete ACL rupture [22, 23]. Furthermore, another previous studies reported that conservative therapy (e.g., using specific brace) showed a significant improvement of anterior knee laxity comparable to patients treated with ACL reconstruction [21, 25]. These previous studies pointed out that it is important to move articulation closer to normal and move it for protecting from early phase after injury. In addition, previous studies have shown that controlling abnormal joint movement in a completely ruptured ACL results in the down-regulation of inflammatory responses [33] and leads to spontaneous healing [27]. Furthermore, importance of weight-bearing has been pointed out in previous study [36]. The results of the present study also showed that an ACL remnant could be maintained by controlling abnormal joint movement. Therefore, controlling abnormal joint movement and weight-bearing may contribute to a spontaneous healing response of the ACL. Furthermore, in the case of ACL reconstruction, the remnant is a very important factor. Previous studies found that the preservation of the remnant tissue improved revascularization and remodeling of the graft and enhanced the biomechanical properties of the graft [37–41]. Consequently, controlling abnormal joint movement and weight-bearing after ACL injury is important not only for conservative therapy but also for patients who undergo ACL reconstruction.

Recent studies have focused on inflammation of the intra-articular tissues after ACL injury [17, 31]. The coordinated expression of MMP-13 in intra-articular tissues and its accumulation in the synovial fluid may lead to excessive matrix degeneration [31]. Moreover, increased MMP-13 expression has been implicated in osteoarthritis and rheumatoid arthritis [42]. Previous studies have also reported that TIMP-1 specifically inhibits MMP-13 [43, 44], and the MMP-13/TIMP-1 ratio is very important for tissue structure. In normal tissues, the stoichiometric MMP-13/TIMP-1 ratio is 1:1 [32]. An increase in MMP-13 is associated with the progression of tissue destruction, and an increase of TIMP-1 is associated with the progression of tissue fibrosis [45]. Compared to the IN and SO groups, the ACL-T and CAM groups showed significantly higher expression levels of MMP-13 and TIMP-1 mRNA and a higher MMP-13/TIMP-1 ratio in all intra-articular tissues. Furthermore, the ACL-T group showed significantly higher MMP-13 mRNA expression compared with the CAM group. Thus, the results suggest that controlling abnormal joint movement after ACL injury inhibited MMP-13 mRNA expression in the intra-articular tissues. Tang et al. reported that MMP-13 mRNA is expressed in the ACL and the intra-articular tissues during the acute phase of ACL injury [31]. The present study similarly showed that the MMP-13 mRNA expression levels were significantly higher in the intra-articular tissues after the acute phase of ACL injury. The ACL is located within the articular capsule, and after injury, the ACL is exposed to synovial fluid containing inflammatory substances that inhibit healing [46]. Previous studies have also reported that the expression of MMP-13 mRNA significantly increases in the cartilage and synovium after ACL injury [31, 47, 48]. Therefore, it is thought that MMP-13 is expressed cooperatively by other intra-articular tissues, accumulates in the synovial fluid, and may promote ACL degeneration after injury. Accordingly, inhibiting the expression of MMP-13 in the intra-articular tissues is considered an important factor in inhibiting the degeneration of articular tissue.

The CAM group showed significantly lower MMP-13 mRNA expression in the intra-articular tissues than did the ACL-T group. The differences in these groups was the use suppression of abnormal movement of the tibia relative to the femur after ACL injury affected our results. Moreover, we showed that the mRNA expression levels of TIMP-1, an inhibitor of MMP-13, increase as the levels of MMP-13 mRNA increase, thereby inhibiting the activity of MMP-13 [49]. Because the expression of TIMP-1 mRNA in the intra-articular tissues was not significantly different between the ACL-T and CAM groups, the ACL-T group showed a significantly higher MMP-13/TIMP-1 ratio than the CAM group on day 5. Thus, MMP-13 and TIMP-1 were not balanced in the ACL-T group. Moreover, a previous study also indicated that degeneration, such as osseous tissue deterioration, results from the increased expression of MMP relative to that of TIMP [50]. Therefore, more tissue degeneration was assumed to have occurred in the ACL-T group than in the CAM group based on the dynamics of MMP-13 and TIMP-1.

Previous studies have reported that meniscus injury can occur due to mechanical stress from abnormal movement of the tibia after ACL injury [2, 50]. Allen et al. reported increases in the front drawer of the tibia and the amount of the load response to the medial meniscus after ACL injury [51]. Levy et al. reported that the medial meniscus contributes to the braking of the tibial front drawer after ACL injury [52]. Therefore, mechanical stress to the medial meniscus increases after ACL injury. The lateral meniscus is an important component in the braking of the tibial front drawer. Musahl et al. reported that the amount of forward displacement significantly increased compared to the amount of displacement prior to excision of the lateral meniscus [53]. Therefore, both the medial meniscus and the lateral meniscus have the ability to brake the tibial front drawer. However, the medial and lateral menisci repeatedly receive mechanical stress from abnormal joint movement after ACL injury. Degeneration of the medial and lateral menisci occurs after an ACL injury due to this repeated secondary mechanical stress. In the present study, the MMP-13 mRNA expression of the medial meniscus and lateral meniscus was higher in the ACL-T group than in the CAM group due to this secondary mechanical stress.

The ACL is responsible for braking the forward movement of the tibia relative to the femur and for tibial rotation. After ACL injury, this braking performance deteriorates, and the loss of this braking capacity in ACL injury patients causes repeated damage to the articular cartilage and meniscus. A previous study reported that more than half of patients with an ACL injury suffer from secondary knee OA [54]. The results of the present study suggested that the expression of MMP-13 mRNA in the acute phase of ACL injury was inhibited by controlling abnormal joint motion. This finding indicates a possible prevention strategy for joint degeneration after ACL injury. A previous study reported that the occurrence of additional knee injuries increased over time after ACL injury, and the risk of additional meniscus injuries increased substantially 6 months after ACL injury [55]. ACL reconstruction surgery is therefore recommended within 6 months after injury, but our findings suggest that degeneration of the joint has already occurred during the acute phase of ACL injury. Consequently, joint control during the acute phase is very important for patients with reconstruction therapy after ACL injury.

The present study has several limitations. First, the investigation was a small animal study, which limits the generalizability of the results. The anatomical components of humans and rats are similar; however, the joint function of the knee and weight-bearing conditions are different. These differences affect the joint kinematics of the knee after ACL injury. Thus, the effect of controlled abnormal joint movement in the early phase of complete ACL rupture may differ between humans and rats. Furthermore, although we already performed the same surgical procedure in rabbit ACL, we cannot confirm for healing ACL in the rabbit. We considered that the difference in walking style between rabbits and rats is affecting. Although rats walk for four legs, walk alternately like humans. On the other hands, rabbit moves forward by kicking the ground with both legs. Therefore, it is necessary to select animals with four leg walking (e.g., pig and/or dog) in future research. Second, we studied the healing process of a completely injured ACL only during the early phase (i.e., until post-injury day 5). Third, this study was performed a small sample. Therefore, statistical differences could not be considered in histologic examination. In the future, it is necessary to quantitatively present the narrowing of the ACL stump by increasing the number of samples. Fourth, in the molecular biology evaluation of the present study, we examined only MMP-13 and TIMP-1. Research has shown that inflammatory factors such as cytokines (e.g., interleukin (IL)-6 and IL-8) are present at elevated levels in synovial fluid during the acute phase of ACL injury [28]. Therefore, it will be ideal to evaluate inflammatory factors in addition to MMPs and TIMPs. Finally, our method for rupturing the ACL was unlike the common mechanism of ACL injury in humans. Further long-term studies using different animals, other inflammatory factors, and another method of ACL rupture are needed to clearly understand the mechanism of spontaneous ACL healing.

## Conclusion

We investigated the effects of changes in the intra-articular environment on the spontaneous healing of an ACL injury during the acute phase using a spontaneously healing rat model. The present study showed the following: (1) after cleavage of the ligament tissue in the CAM group, degeneration of the ligament was not observed compared with the ACL-T group, and (2) the CAM group showed significant reductions in MMP-13 mRNA expression and the MMP-13/TIMP-1 ratio compared with that in he ACL-T group on day 5. These results suggest that controlling abnormal movement inhibits the inflammatory reaction in the intra-articular tissues after ACL injury.

## Abbreviations

ACL: Anterior cruciate ligament
ACL-T: ACL-transection
ANOVA: Analysis of variance
CAM: Controlled abnormal movement
cDNA: Complementary DNA
H&E: Hematoxylin and eosin
IN: Intact
MCL: Medial collateral ligament
MMP-13: Matrix metalloproteinase-13
PRC: Reverse transcription polymerase chain reaction
SD: Standard deviation
SO: Sham-operated
TIMP-1: Tissue inhibitor of metalloproteinase-1
